# The pregnant women's perception of risks and pregnancy stress levels: a cross-sectional study from Turkey

**DOI:** 10.1590/1806-9282.20231270

**Published:** 2024-07-19

**Authors:** Aytan Maharramova, Elif Yagmur Gur

**Affiliations:** 1 Liv Bona Dea Hospital – Baku, Azerbaijan.; 2 Eskisehir Osmangazi University, Health Science Faculty, Department of Midwifery – Eskişehir, Turkey.

**Keywords:** Pregnancy, Stress

## Abstract

**OBJECTIVE:**

The aim of this study was to compare pregnant women's perceptions of risk and pregnancy-specific stress levels.

**METHODS:**

This cross-sectional descriptive study was conducted with 410 healthy pregnant women at the city hospital located in the east of Turkey. Data were collected via Personal Information Form, Perception of Pregnancy Risk Questionnaire, and Pregnancy Stress Rating Scale.

**RESULTS:**

The pregnancy risk perception mean score was 2.43±1.82, and the pregnancy-specific stress mean score was 22.27±12.67. There is a statistically significant and strong positive correlation between the perception of pregnancy risk and pregnancy-specific stress level (p<0.01). Pregnant women's pregnancy risk perception decreased as the duration of marriage and the number of living children increased, and it increased as the gestational week increased (p<0.05). Pregnancy-specific stress decreased as the duration of marriage (p<0.001), the age of the spouse, the number of pregnancies, and the number of living children increased (p<0.01), and it increased as the gestational week increased (p<0.01).

**CONCLUSION:**

The pregnant women's perceptions of pregnancy risks and pregnancy-specific stress were low, but pregnancy-specific stresses increased as their perceptions of pregnancy risks increased.

## INTRODUCTION

The concept of pregnancy-related risk perception can affect women's emotional state and decision-making about pregnancy and childbirth process^1^. Also, pregnancy-specific stress is briefly defined as a woman's concerns, anxiety, and fear about pregnancy^2^. Pregnancy-related stress and its adverse effects^3-6^ are considered to be one of the leading causes of maternal perinatal deaths^3^. In addition, pregnancy-specific stress may be creating adverse effects on the fetus regardless of obstetric risks^5^, such as risks for low birth weight, premature birth^7,8^, and fetal developmental disorders^6^. Therefore, it is important to identify women who suffer from psychological stress during pregnancy. The American Society of Gynecology and Obstetrics recommends prenatal screening and intervention for psychosocial risk factors, including stress, in all pregnant women^4^. Health care professionals could collaborate in determining the perception of pregnancy risks and pregnancy-specific stress and identifying and implementing effective strategies to manage this process^9^. Considering the importance of the topic, it aimed to determine the correlation between the women's perceptions of pregnancy risk and pregnancy-specific stress levels in this study.

## METHODS

### Study sample and design

This descriptive cross-sectional study was carried out according to STROBE guidelines. It was conducted in a city hospital in a province in eastern Turkey between December 2021 and March 2022. The study involved 11,623 women, who came to the hospital for routine pregnancy check-ups in 2021. Using the known sampling formula, the study sample was calculated as 372, and it was completed with 410 pregnant women.

### Inclusion criteria

Inclusion criteria were as follows: healthy pregnant women, aged between 18 and 35 years, could speak Turkish language, were Turkish citizens, and did not have any physical/mental health problems/pregnancy risks.

### Data collection

The data were collected using the Personal Information Form, Perception of Pregnancy Risk Questionnaire, and Pregnancy Stress Rating Scale. The interviews were held face to face, and the duration was about 10–15 min.

### Data collection tools

Personal Information Form: This form consisted of 17 questions about sociodemographic and obstetric characteristics. Perception of Pregnancy Risk Questionnaire (PPRQ): The questionnaire consists of nine items and two factors. Each item on the scale has a 0–10-cm long line (0–100 mm), with the left extreme reading "no risk" and the other extreme "extremely high risk." The total score of the scale is obtained by summing the scores of the items and dividing the result by nine. The scale has no cutoff point. High total scores on the scale indicate that the risk perception of the pregnant woman about herself and her child increases^10,11^. In the current study, Cronbach's alpha value was found to be 0.80.

Pregnancy Stress Rating Scale (PSRS): This scale has 36 items and a 5-point Likert type. The sum of item scores gives the prenatal stress score. The score obtained from the scale is between 0 and 144. High total scores indicate an increase in perceived prenatal stress^12,13^. In the current study, Cronbach's alpha value was found to be 0.83.

### Data analysis

The study was analyzed using the SPSS program (Statistics Package for Social Sciences for Windows, Version 21.0, IBM Corp., NY) using the counts, percentages, mean scores, one-way ANOVA, Kruskal-Wallis, Mann-Whitney U test, independent samples t-test, and Pearson correlation test. A p-value of <0.05 was considered significant for all statistical tests.

### Ethical approval

The approval of the Ethics Committee of University Faculty of Medicine and the written permission of the hospital were obtained. It was conducted in compliance with the principles of the Declaration of Helsinki.

## RESULTS

The mean age of the pregnant women was 25.75±4.05 years, the mean age of the spouses was 30.18±4.45 years, the mean duration of marriage was 4.09±3.40 years, and the mean gestational week was 28.49±7.05. Number of pregnancy was 1.85±1.13 and number of living children was 0.62±0.87 (data not shown).

A statistically significant difference was found between the descriptive characteristics of the pregnant women, namely, duration of the marriage, education level of the spouse, and parity and their mean scores on the total PPRQ and PSRS (p<0.05, Table 1).

**Table 1:** Comparison of participants' Perception of Pregnancy Risk Questionnaire and Pregnancy Stress Rating Scale mean scores with their descriptive characteristics.

Variables		n	%	PPRQ		PSRS	
				$\bar{\text{x}}\pm \text{SD}$	Test and p-value	$\bar{\text{x}}\pm \text{SD}$	Test and p-value
Age							
	18–23	130	31.7	2.39±1.77	F=0.56 p=0.57	22.32±12.51	F=3.72 **p=0.02**
	24–29	189	46.1	2.53±1.87		23.67±13.45	
	30–35	91	22.2	2.29±1.80		19.27±10.68	
Education							
	Primary education	116	28.3	2.17±1.83	F=2.77 p=0.06	20.92±12.55	F=2.54 p=0.08
	High school	165	40.2	2.40±1.68		21.63±12.21	
	University	129	31.5	2.71±1.96		24.30±13.19	
Marriage duration							
	1–5 years	306	74.6	2.61±1.83	KW=13.57 **p=0.001**	23.62±12.66	KW=17.56 **p=0.0001**
	6–10years	79	19.3	1.93±1.79		19.50±12.58	
	≥11 years	25	6.1	1.84±1.46		14.40±8.30	
Partner's education							
	Primary education	84	20.5	2.06±1.79	F=3.95 **p=0.02**	19.71±13.01	F=4.64 **p=0.01**
	High school	170	41.5	2.35±1.70		21.45±11.37	
	University	156	38.0	2.72±1.93		24.53±13.50	
Family type							
	Nucleus	346	84.4	2.45±1.78	t=0.61 p=0.54	22.52±12.62	t=0.92 p=0.35
	Large	64	15.6	2.30±2.06		20.92±12.94	
Social support							
	Yes	348	84.9	2.38±1.82	t=1.27 p=0.20	21.56±12.59	t=2.69 **p=0.007**
	No	62	15.1	2.70±1.85		26.24±12.45	
Planned pregnancy							
	Yes	343	83.7	2.39±1.84	t=1.03 p=0.30	21.86±12.64	t=1.48 p=0.13
	No	67	16.3	2.64±1.70		24.37±12.69	
Parity							
	Primiparous	218	53.2	2.62±1.83	t=2.29 **p=0.02**	23.44±12.82	t=1.99 **p=0.04**
	Multiparous	192	46.8	2.21±1.79		20.94±12.38	

Findings about the relationships between some descriptive variables of the study and PPRQ and PSRS are given in Table 2. There was a negative correlation between the mean PPRQ score of the pregnant women, duration of the marriage, and the number of living children (r=-0.125, p=0.011; r=-0.169, p=0.001, respectively) and a statistically significant positive correlation with the gestational week (r=0.126, p=0.011). It was determined that there was a statistically significant difference between pregnant women's age groups, perception of social support, and their mean PSRS scores (p<0.05). There was a negative correlation between pregnant women's characteristics, namely, the age of the spouse, duration of the marriage, parity, the number of living children, and their mean score on the total PSRS (r=-0.129, p=0.009; r=-0.185, p=0.000; r=-0.143, p=0.004; r=-0.192, p=0.000, respectively) and a statistically significant positive correlation with the gestational week (r=0.153, p=0.002).

**Table 2:** Correlation of some descriptive variables with Perception of Pregnancy Risk Questionnaire and Pregnancy Stress Rating Scale.

		PPRQ	PSRS
Age	r	-0.038	-0.072
	p	0.440	0.146
Partner age	r	-0.059	-0.129**
	p	.234	**0.009**
Marriage duration	r	-0.125*	-0.185**
	p	**0.011**	**0.000**
Gestational weeks	r	0.126*	0.153**
	p	**0.011**	**0.002**
Number of pregnancy	r	-0.056	-0.143**
	p	0.261	**0.004**
Number of living children	r	-0.169**	-0.192**
	p	**0.001**	**0.000**

* p<0.05.

** p<0.01.

It was determined that the PPRQ mean score of the pregnant women was 2.43±1.82 and 22.27±12.67 on the total PSRS. There was a statistically significant positive correlation between the mean scores of the pregnant women on the total PPRQ and the PSRS (p<0.01, Table 3).

**Table 3:** The relationship between the Perception of Pregnancy Risk Questionnaire and Pregnancy Stress Rating Scale mean scores of study participants.

Scales	Theoretical Min–Max value	Received Min–Max value	$\bar{\text{x}}\pm \text{SD}$	r	p
PPRQ	0–10	0–9.44	2.43±1.82	0.662**	0.000
PSRS	0–144	0–62.00	22.27±12.67		

** p<0.01.

## DISCUSSION

The study indicated that both the pregnancy risk perception and the pregnancy-specific stress score averages of pregnant women were low. The fact that the majority of women had social support and had a planned pregnancy may have affected this result. Also, some studies report similar findings as the current study^14,15^.

In this study, as the risk perception of pregnant women increased, their pregnancy-specific stresses increased as well. Similar to this finding, some studies in the literature show that stress had a significant effect on the perception of pregnancy risk^14,16^. It is thought that women's thoughts about possible harm to themselves and their babies could have increased the perception of pregnancy risk.

In this study, women's perceptions of pregnancy risk and pregnancy-related stress were highest in the 24–29 age group, and it was also observed that pregnancy-specific stress decreased significantly as the age of the pregnant women's spouses increased. The age of the spouse can also influence pregnancy-specific stress levels. However, an adult and experienced partner could help reduce/control pregnancy stress by positively affecting the psychological adjustment of the pregnant woman^17^. In the current study, as the duration of the marriage of pregnant women decreased, perceptions of pregnancy risks increased. Pregnant women who are newly married or have a short marriage period may not be able to adapt psychologically to pregnancy, which is an important period of life, as they may not have had enough time to adapt to their spouse, family, and new living environment^18^. In addition, as the duration of the marriage increased in this study, their pregnancy-specific stress decreased. Similarly, one study reported that pregnancy-specific stress decreased as the duration of the marriage increased^4^. The increase in the duration of the marriage may pave the way for the formation of planned pregnancies by boosting the harmony between couples, marital harmony, and social support. In this study, the majority of pregnant women had a planned pregnancy, which may have facilitated their adaptation to pregnancy and may have reduced pregnancy-specific stress.

The current study has shown that as the education level of the spouses of pregnant women increased, the perceptions of pregnancy risks and pregnancy-related stress increased as well. Increasing the level of consciousness may cause excessive focus on the healthy process of pregnancy in spouses. Another reason that increases pregnancy stress may be the increase in the control of the spouse over the pregnancy and the decrease in the self-control of the pregnant woman, which is supported by a previous study^19^. In this study, it was seen that the pregnancy-specific stress of pregnant women receiving social support was statistically significantly lower than the stress of those with no social support. Staneva et al.^20^ reported similar findings as that of the current study. It has been emphasized that a decrease in pregnant woman's personal–social factors increases her stress level^21^.

In the current study, the perceptions of pregnancy risks and pregnancy-specific stress of multiparous women were less than those of primiparous. This result can be interpreted as follows. Multiparous pregnant women have more experience with pregnancy than primiparous women. Having experience with an event means that perceived risk will be generally lower when encountering the same event. Low levels of pregnancy-specific stress can be explained by previous pregnancy experience and adaptation to stressors. Similar to the findings of this study, some studies have shown that multiparous pregnant women's stress is significantly lower than that of the primiparous^17,19^. Another remarkable parameter in the study was the gestational week. As the gestational week progressed, pregnant women's risk perception and pregnancy-specific stress increased. The progressing gestational week or the upcoming delivery may cause an increase in women's concerns about delivering their child healthily and providing a good future for it. It has been reported that such concerns are effective in the perception of pregnancy risks^14^. In the study, as having living children increased, perceptions of pregnancy risks and pregnancy-specific stress decreased. As the survival rate of children born by a woman increases, the experience can shape her psychological state positively. It is thought that the finding of this study may be related to this situation.

## CONCLUSION

In this study, it was found that pregnant women had a low perception of pregnancy risks and pregnancy-specific stress. It was also observed that pregnancy-specific stress increased as their perceptions of pregnancy risks increased. Further research, including prospective studies with different sample groups and influencing factors, is needed to elucidate the relationship between pregnancy risk perception and pregnancy-specific stress.
